# Targeting ALDH1A1 by disulfiram/copper complex inhibits non-small cell lung cancer recurrence driven by ALDH-positive cancer stem cells

**DOI:** 10.18632/oncotarget.11305

**Published:** 2016-08-16

**Authors:** Xinwei Liu, Lihui Wang, Wei Cui, Xiangzhong Yuan, Lulu Lin, Qi Cao, Nannan Wang, Yi Li, Wei Guo, Xun Zhang, Chunfu Wu, Jingyu Yang

**Affiliations:** ^1^ Department of Pharmacology, Shenyang Pharmaceutical University, Shenyang, P.R. China; ^2^ Benxi Institute of Pharmaceutical Research, Shenyang Pharmaceutical University, Benxi, P.R. China

**Keywords:** disulfiram/copper, ALDH1A1, cancer stem cell, NSCLC, recurrence

## Abstract

The existence of cancer stem cells (CSCs) in non-small cell lung cancer (NSCLC) has profound implications for cancer therapy. In this study, a disulfiram/copper (DSF/Cu) complex was evaluated *in vitro* and *in vivo* for its efficacy to inhibit CSCs, which drive recurrence of NSCLC. First, we investigated whether DSF/Cu could inhibit ALDH-positive NSCLC stem cells *in vitro* and tumors derived from sorted ALDH-positive CSCs *in vivo*. DSF/Cu (0.5/1 μmol/l) significantly inhibited the expression of stem cell transcription factors (Sox2, Oct-4 and Nanog) and reduced the capacities of NSCLC stem cells for self-renewal, proliferation and invasion *in vitro*. Regular injections with DSF/Cu (60/2.4 mg/kg) reduced the size of tumors derived from sorted ALDH-positive stem cells. Two other NOD/SCID xenograft models were used to determine whether DSF/Cu could target NSCLC stem cells and inhibit tumor recurrence *in vivo*. DSF/Cu treatment eliminated ALDH-positive cells and inhibited tumor recurrence, which was reflected by reduced tumor growth in recipient mice that were inoculated with tumor cells derived from DSF/Cu-treated cells or primary xenografts. RNA interference and overexpression of ALDH isozymes suggested that ALDH1A1, which plays a key role in ALDH-positive NSCLC stem cells, might be the target of the DSF/Cu complex. Collectively, our data demonstrate that DSF/Cu targets ALDH1A1 to inhibit NSCLC recurrence driven by ALDH-positive CSCs. Thus, the DSF/Cu complex may represent a potential therapeutic strategy for NSCLC patients.

## INTRODUCTION

The cancer stem cell model proposes that tumor progression, drug resistance, metastasis, and relapse after therapy may be driven by a subset of cells within a tumor. This phenomenon is a functionally important example of intra-tumor heterogeneity [1, 2]. During the past few years, cancer stem cells (CSCs) have been identified in, and isolated from, solid tumors such as breast, brain, colon, pancreatic, and prostate tumors [3–7]. The identification of CSCs has important implications for future therapeutic approaches. Recent evidence suggests that like other tumors, human lung cancers may also harbor CSC populations [8, 9]. However, identification and targeting of human lung CSCs has been hampered by the lack of reliable lung cancer stem cell markers [10].

The aldehyde dehydrogenase (ALDH) family comprises cytosolic isoenzymes responsible for oxidizing intracellular aldehydes, including the oxidation of retinol to retinoic acid in early stem cell differentiation [11]. Recent evidence suggests that ALDH activity has also identified CSCs in a variety of tumor types [12–22]. Disulfiram (DSF) is an aldehyde dehydrogenase inhibitor that was used as a vermicide in the 1930s and for alcohol aversion therapy since the 1940s [11]. Accumulating evidence demonstrates that DSF has strong anticancer activity against certain types of cancer both *in vitro* and in mouse models [23–26]. Inhibition of ALDH activity has been investigated as a potential strategy to eliminate cancer stem cells [27], and the results suggest that DSF may specifically target cancer stem cell subpopulations. Also, previous studies indicated that the cytotoxicity of DSF is copper (Cu)-dependent [26, 28–30]. In breast cancer, DSF and copper treatment inhibits NF-κB activity, elevates ROS levels and decreases the number of breast cancer stem cells *in vitro* [31]. DSF, either alone or in combination with copper, inhibits the growth of lung cancer cells *in vitro* [32]. These findings led us to investigate the effect of DSF/copper complex treatment on ALDH-positive NSCLC stem cells *in vitro* and *in vivo*.

In this study, we examined the efficacy of the DSF/Cu complex against ALDH-positive NSCLC stem cells in cell lines and lung cancer xenografts. Our *in vivo* data showed that the DSF/Cu complex was more effective than DSF alone at eliminating ALDH-positive cells and inhibiting tumor recurrence, as reflected by the inhibition of tumor growth in recipient mice that were inoculated with tumor cells derived from DSF/Cu-treated cell lines or primary xenografts. Furthermore, we investigated the stem cell-related function and significance of ALDH isozymes in NSCLC cell lines. Our data showed that ALDH1A1, which plays a key role in ALDH-positive NSCLC stem cells, is the target of the DSF/Cu complex.

## RESULTS

### ALDH-positive cells represent cancer stem cells in NSCLC cell lines

**Figure 1:**
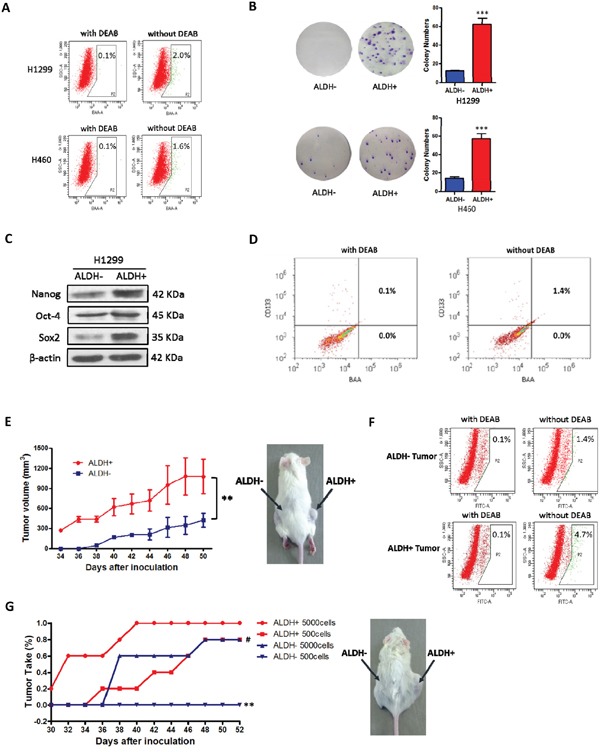
ALDH-positive cells represent cancer stem cells in some NSCLC cell lines **A.** ALDH-positive and ALDH-negative cells were isolated from the indicated NSCLC cell lines by FACS. Brightly fluorescent ALDH-expressing cells (ALDH-positive cells) were detected in the green fluorescence channel (BAA) using flow cytometry. DEAB, a specific inhibitor of ALDH, was used to establish the baseline fluorescence of these cells and to define the ALDH-positive region. **B.** Analysis of cell colony numbers in colony forming assays of ALDH-positive and ALDH-negative cells (***P < 0.001, χ^2^ test). **C.** Analysis of stem cell transcription factors by western blotting. **D.** Double staining of Aldefluor and CD133 (PE) in NCI-H1299 cells. **E.** Comparison of primary xenograft formation by sorted ALDH-positive and ALDH-negative NCI-H1299 cells in NOD/SCID mice (**P < 0.01, 2-tailed *t* test). **F.** The percentage of ALDH-positive cells in xenograft tumors derived from ALDH-positive and ALDH-negative cells. **G.** Comparison of tumor take (%) in NOD/SCID mice with secondary xenografts of ALDH-positive and ALDH-negative cells taken from primary xenograft tumors (**P < 0.01, χ^2^ test, compared with the ALDH+ 500 cells group; #*P* < 0.05, χ^2^ test, compared with the ALDH+ 5000 cells group).

ALDH activity can be determined by the Aldefluor assay, which has been used to identify CSCs in a variety of tumor types [12–22]. Although previous reports have characterized the ALDH-positive CSC population in some NSCLC cell lines [21, 22], it is necessary to confirm the identity of CSCs in specific experimental environments. Therefore, Aldefluor assays followed by FACS analysis were used to assess the presence of a cell population with ALDH activity, and then colony forming assays were used to compare the colony forming capacity of ALDH-positive and ALDH-negative cells in four NSCLC cell lines (NCI-H1299, NCI-H460, NCI-H522 and A549). We found that all cell lines had a small ALDH-positive population, with 2.0% (2.00 ± 0.03) in NCI-H1299, 1.6% (1.60 ± 0.67) in NCI-H460, 1.9% (1.87 ± 0.04) in NCI-H522 and 0.2% (0.23 ± 0.04) in A549 (Figure 1A and Supplemental Figure S1). However, only in the NCI-H1299 and NCI-H460 cell lines did the ALDH-positive cells show a significantly higher colony-forming efficiency than the ALDH-negative cells, as judged by clonal assays (Figure 1B and Supplemental Figure S1). These data indicated that the ALDH-positive subpopulation of the NCI-H1299 and NCI-H460 cell lines possessed a high self-renewal capacity. We also found that some other NSCLC cell lines, such as A549, contain cells with high colony forming efficiency that are positive for other putative CSC markers, such as CD133 (Supplemental Figure S2). Therefore, the NCI-H1299 and NCI-H460 cell lines were chosen as models for further research into the role of ALDH in CSCs.

Oct4, Sox2 and Nanog are consistently detected in human CSCs [33], so we compared their expression in ALDH-positive and ALDH-negative cells. Our data showed that Nanog, Oct-4 and Sox2 were expressed at higher levels in ALDH-positive NCI-H1299 cells than in ALDH-negative NCI-H1299 cells, suggesting that in the NCI-H1299 cell line, ALDH expression may be essential for maintaining self-renewal and tumorigenesis (Figure 1C). To further explore whether ALDH is a single CSC marker in NSCLCs, H1299 cells were double-stained with Aldefluor and another putative NSCLC stem cell marker, CD133 (PE). As shown in Figure 1D, the whole ALDH-positive population (1.4%) also showed a high level of CD133 expression. The proportion of ALDH+/CD133- cells was 0.0%. There was a high degree of overlap between CD133 and ALDH expression.

The gold standard for identification of CSCs is whether the cells can preferentially initiate tumor development in animal models [2]. To investigate possible differences in tumor-forming potential between the ALDH-positive and ALDH-negative sorted cells, 5×10^3^ ALDH-positive and 5×10^3^ ALDH-negative NCI-H1299 cells were injected under the skin in opposite sides of NOD/SCID mice (4 mice in each group). After 7 weeks, the ALDH-positive NCI-H1299 cells generated much larger tumors than the ALDH-negative cells in all the mice (Figure 1E). To elucidate whether ALDH-positive H1299 cells could create larger tumors with heterogeneity *in vivo*, Aldefluor analysis was carried out on disassociated cells from the first generation tumor engrafts. The engrafted tumors derived from ALDH-positive cells gave rise to 4.7% ALDH-positive cells and the tumors derived from ALDH-negative cells gave rise to 1.4% ALDH-positive cells (Figure 1F). Living cells from the dissociated tumor engrafts were sorted for secondary xenografts. ALDH-positive and ALDH-negative cells were inoculated in opposite sides of NOD/SCID mice at a dose of 5,000 or 500 cells. The results showed that the secondary xenograft tumors that developed from the ALDH-positive cells were larger than the tumors from ALDH-negative cells, especially in the 500 cells group (Figure 1G). Our *in vivo* data suggested that ALDH-positive NCI-H1299 cells possess the unique features of cancer stem-like cells, including initiation of tumorigenesis, self-renewal, and the reinitiation of serially transplantable tumors.

Taken together, the results of the *in vivo* and *in vitro* assays indicated that within some NSCLC cell lines, the ALDH-positive cells have the broadest self-renewal and differentiation potential *in vitro* and the highest growth potential *in vivo*.

### DSF/Cu inhibits the stemness of NSCLCs *in vitro*

**Figure 2:**
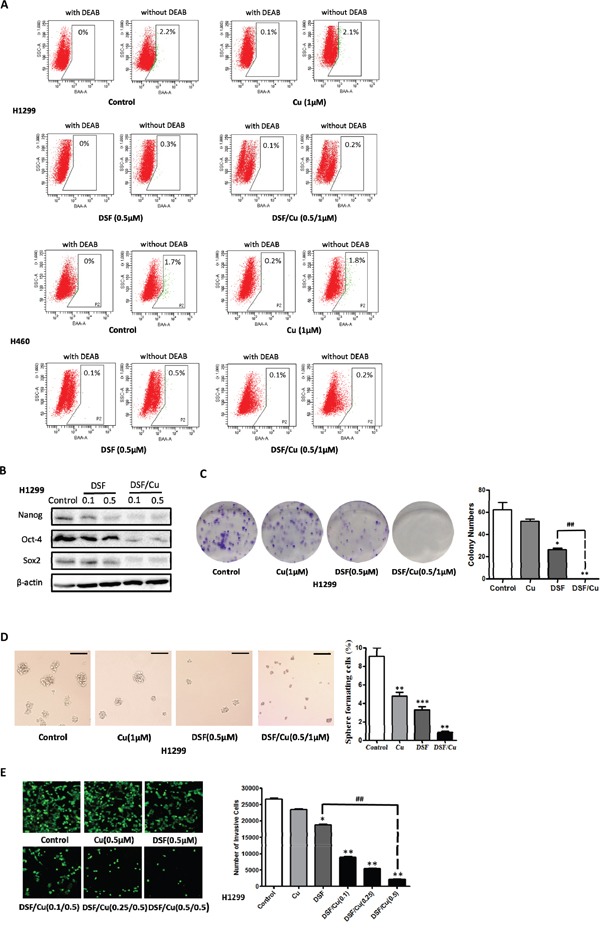
DSF/Cu inhibits the stemness of NSCLCs *in vitro* **A.** Inhibitory effect of DSF (0.5 μM), Cu (1 μM) and DSF/Cu (0.5/1 μM) on the ALDH-positive cell population from the NSCLC cells lines H1299 and H460. **B.** DSF (0.1 μM, and 0.5 μM) and DSF/Cu (0.1/1 μM, and 0.5/1 μM) inhibit the expression of the stem cell transcription factors Nanog, Sox2 and Oct-4 expression in H1299 cells. **C.** Inhibitory effect of DSF (0.5 μM), Cu (1 μM) and DSF/Cu (0.5/1 μM) on the colony forming ability of H1299 cells (*P < 0.05, **P < 0.01, ##*P* < 0.01, one-way ANOVA). **D.** Inhibitory effect of DSF and DSF/Cu on tumorsphere formation. Tumorspheres were incubated with DSF (0.5 μM), Cu (1 μM) and DSF/Cu (0.5/1 μM) for 7 days. Scale bar, 100 μm. (**P < 0.01, ***P < 0.001, one-way ANOVA). **E.** Inhibitory effect of DSF/Cu on transwell invasion assay. Cells were incubated with DSF (0.5 μM), Cu (0.5 μM) and DSF/Cu (0.1/0.5 μM, 0.25/0.5 μM and 0.5/0.5 μM) in transwells for 24 hours (*P < 0.05, **P < 0.01, one-way ANOVA).

As shown above, ALDH-positive cells represent the stem cell population in some NSCLC cell lines. Therefore, DSF, which has been proved to be an irreversible inhibitor of ALDHs, may also inhibit NSCLC stem cells. Firstly, we examined the effects of DSF and DSF/Cu on the ALDH-positive populations in NCI-H1299 and NCI-H460 cells. As shown in Figure 2A and Supplemental Figure S3, both DSF (0.02 μM, 0.1 μM and 0.5 μM) alone and the DSF/Cu complex (0.02/1 μM, 0.1/1 μM and 0.5/1 μM) significantly reduced the proportion of ALDH-positive cells (control: 2.03% ± 0.38%; DSF, 0.5 μM: 0.33% ± 0.04%; DSF/Cu, 0.5/1 μM: 0.07% ± 0.04%).

Next, we examined the inhibitory effects of DSF, Cu and DSF/Cu treatment on the expression of the stem cell transcription factors Nanog, Sox2 and Oct-4. As shown in Figure 2B, DSF/Cu dramatically reduced the expression of all three stem cell transcription factors in a concentration-dependent manner, and the inhibitory activity of DSF/Cu was far more potent than DSF alone.

We further tested the ability of DSF and DSF/Cu to inhibit the colony-forming efficiency of ALDH-positive cells derived from NCI-H1299 cells. As shown in Figure 2C, DSF/Cu (0.5/1 μM) had a significantly stronger inhibitory effect on colony formation than DSF alone (0.5 μM). We also compared the ability of DSF/Cu to inhibit the colony-forming ability of cell lines which harbor ALDH-positive CSCs and those which do not harbor ALDH-positive CSCs. We examined the efficacy of DSF/Cu on the ALDH-positive subsets in the NCI-H1299, NCI-H460 and A549 cell lines. As shown in Supplemental Figure S5, DSF/Cu (0.5/1 μM) inhibited the colony-forming ability of ALDH-positive cells in all these lung cancer cell lines. However, the inhibitory efficacy in A549 cells (79.5%) was lower than in NCI-H1299 and NCI-H460 cells (100%). Thus, these results demonstrate that DSF/Cu may be useful in the treatment of lung tumors that harbor a subpopulation of ALDH-positive CSCs.

Similar results were also observed in tumorsphere forming assays. It has been shown that cancer stem/progenitor cells are enriched in non-adherent spherical clusters of cells [2]. To evaluate whether DSF and DSF/Cu can inhibit the formation of tumorspheres *in vitro*, we exposed tumorspheres to DSF (0.5 μM), Cu (1 μM) and DSF/Cu (0.5/1 μM) for 24 h. As shown in Figure 2D and Supplemental Figure S6, DSF and DSF/Cu inhibited the formation of tumorspheres. The inhibitory efficacy of copper may be due to ROS formation, which is promoted by copper ions. We also evaluated the anti-proliferative effects of DSF in NCI-H1299 and NCI-H460 cells by MTT assay (Supplemental Figure S4). The data showed that DSF/Cu significantly inhibited the proliferation of ALDH-positive NSCLC stem cells.

The capacity for invasion and metastasis may also be an important property of CSCs [2]. Therefore, the invasion of NCI-H1299 and NCI-H460 cells was measured by transwell assays after treatment with DSF (0.5 μM), Cu (0.5 μM) and DSF/Cu (0.1/0.5 μM, 0.25/0.5 μM and 0.5/0.5 μM) for 24 h. The results showed that DSF/Cu dramatically reduced the fetal bovine serum (FBS)-induced invasion of NSCLCs in a concentration-dependent manner (Figure 2E and Supplemental Figure S7).

Taken together, these results demonstrated that treatment with DSF/Cu, but not DSF alone, significantly inhibited the stemness of ALDH-positive cells *in vitro*.

### DSF/Cu inhibits ALDH-positive NSCLC stem cells *in vivo*

To determine whether DSF/Cu can inhibit ALDH-positive NSCLS stem cells *in vivo*, we used a xenograft model of ALDH-positive NCI-H1299 cells in NOD/SCID mice. ALDH-positive cell populations were analyzed using the Aldefluor assay and sorted by FACS. Tumor cells were implanted into three groups of mice (five mice in each group).

**Figure 3:**
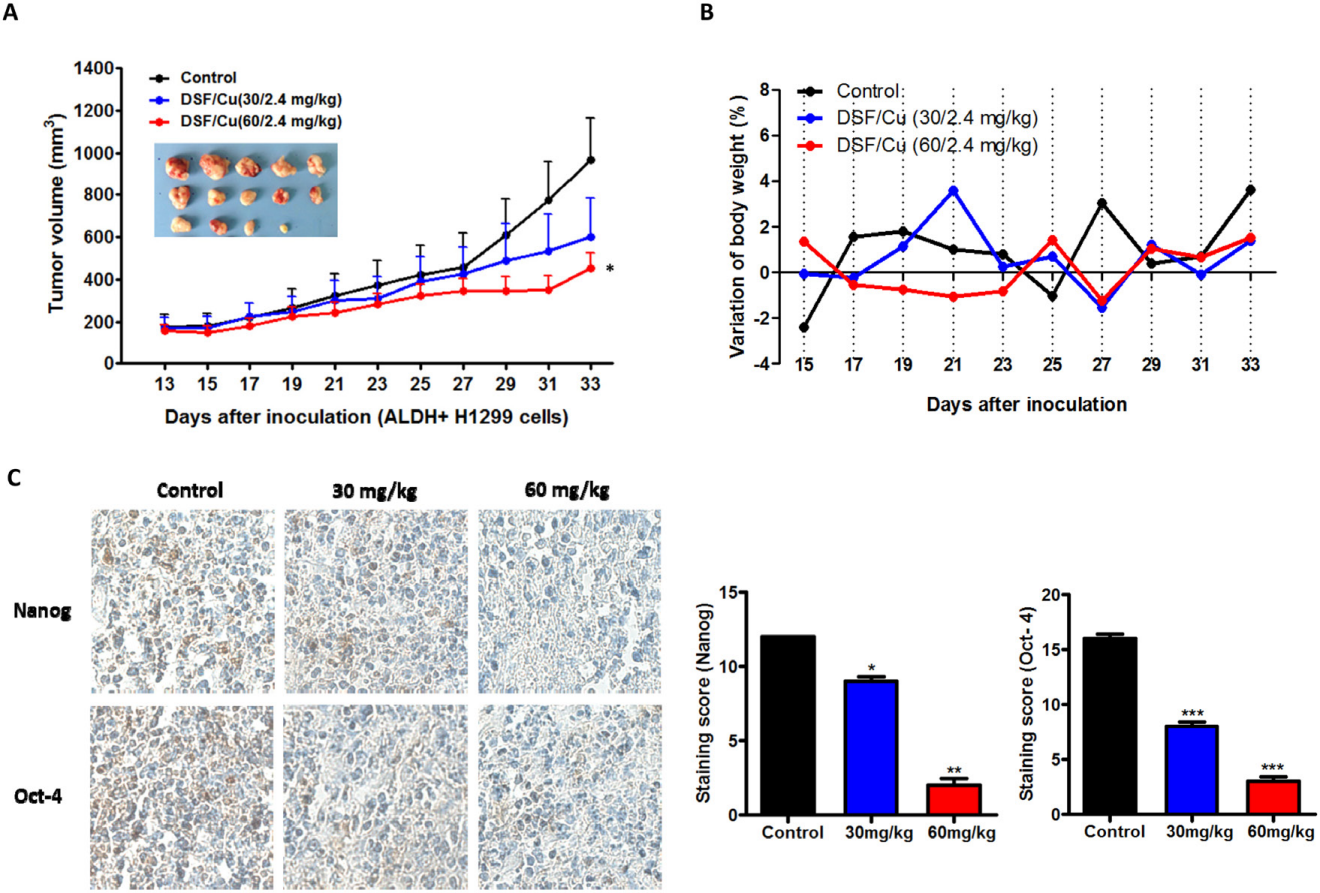
DSF/Cu inhibits ALDH-positive NSCLC stem cells *in vivo* **A.** DSF/Cu decreases the size of tumors derived from ALDH-positive NSCLC xenografts. Images of the tumors that developed in NOD/SCID mice from each treatment group are also shown. (*P < 0.05, one-way ANOVA). **B.** DSF/Cu has no apparent toxicity as determined by body weight. **C.** Representative images of immunohistochemical staining of tumor tissue for the stem cell transcription factors Nanog and Oct-4. The staining intensity was scored as 0 (negative), 1 (weak), 2 (medium) and 3 (strong). Extent of staining was scored as 0 (0%), 1 (1-25%), 2 (26-50%), 3 (51-75%) and 4 (76-100%), according to the percentage of the positively stained areas in relation to the whole carcinoma area (*P < 0.05, **P < 0.01, ***P < 0.001, one-way ANOVA).

Two weeks after cell inoculation, animals were intravenously injected with 0.9% NaCl solution (control) or DSF (DSF-loaded lipid emulsion, 30 mg/kg and 60 mg/kg) with Cu (intragastric administration, 2.4 mg/kg) every two days. After 2 weeks of treatment, tumors in the DSF/Cu (30/2.4 mg/kg) mice were 62.4% of the size of those in the control animals, and tumors in the DSF/Cu (60/2.4 mg/kg) mice were 46.6% of the size of those in the control animals (Figure 3A). DSF/Cu had no apparent toxicity as determined by body weight measurement (Figure 3B). The inhibitory efficacy of DSF/Cu treatment on the expression of the stem cell transcription factors Nanog and Oct-4 in tumor tissues was also examined. As shown in Figure 3C, DSF/Cu treatment inhibited the expression of Nanog and Oct-4 in a dose-dependent manner. These results suggested that DSF/Cu was able to eliminate NSCLC stem cells in xenografted ALDH-positive cells.

### Effects of DSF/Cu treatment on NSCLC stem cell numbers *in vitro* and tumor recurrence *in vivo*

**Figure 4:**
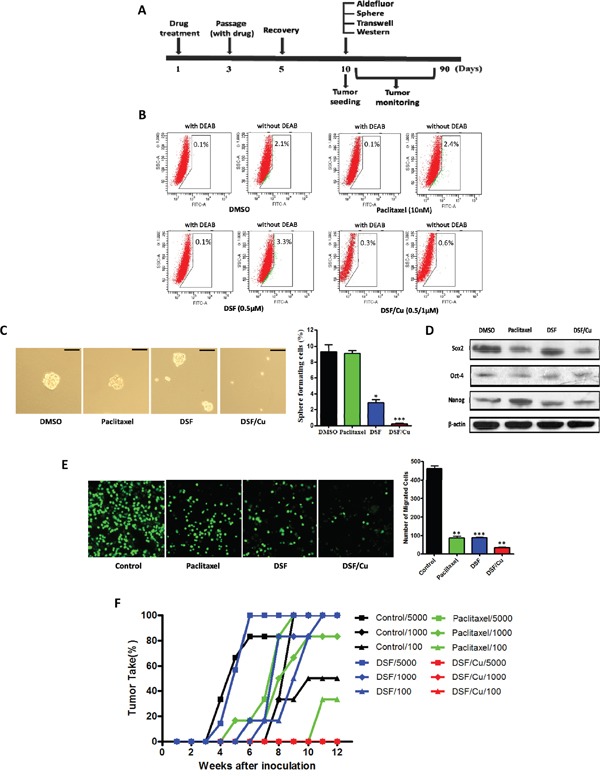
Effects of DSF/Cu pretreatment on NSCLC stem cell numbers *in vitro* and tumor seeding *in vivo* **A.** Schematic representation of treatment scheme. Cells were pretreated with drugs, then allowed to recover before experimental testing. **B.** Inhibitory effect of drug pretreatment on ALDH-positive cell populations before inoculation. **C.** Inhibitory effect of drug pretreatment on tumorsphere formation. Scale bar, 50 μm. (*P < 0.05, ***P < 0.001, χ^2^ test). **D.** Inhibitory effect of drug pretreatment on the expression of stem cell transcription factors. **E.** Inhibitory effect of drug treatment on transwell migration assays (**P < 0.01, ***P < 0.001, one-way ANOVA). **F.** Latency periods and tumor take percentage in xenograft mice receiving cells pretreated with the indicated drugs.

To determine whether DSF/Cu can inhibit tumor regrowth *in vivo*, we assessed the functional presence of CSCs by assaying for *in vivo* tumor-seeding ability after drug treatment *in vitro*. In these experiments, NCI-H1299 cells were treated with drugs *in vitro* for 5 days, allowed to recover and expand in culture for at least 5 days in the absence of drug treatment, then injected in serial limiting dilutions into mice. At the same time, we tested these pretreated cells for ALDH activity, expression of stem cell transcription factors, and proliferation and migration capacity (Figure 4A). As shown in Figure 4B, DSF/Cu significantly decreased ALDH activity (0.3%) relative to the DMSO control (2.0%), but paclitaxel (2.3%) and DSF alone (3.2%) treatment slightly increased the ALDH activity in pretreated cells. These results suggest that in the groups pretreated with paclitaxel and DSF alone, the expansion of ALDH-positive CSCs increased during the recovery process. As shown in Figure 4C, DSF/Cu pretreatment resulted in a >40-fold decrease in sphere-forming ability relative to control or paclitaxel pretreatment. Similarly, DSF/Cu pretreatment inhibited the expression of the stem cell transcription factors Sox2 and Nanog more strongly than DSF alone or paclitaxel (Figure 4D). There was no obvious inhibition of Oct-4 expression. As shown in Figure 4E, paclitaxel, DSF and DSF/Cu pretreatments all inhibited the migratory capacity of the cells, but DSF/Cu pretreatment had enhanced inhibitory efficacy relative to pretreatment with paclitaxel and DSF alone. Taken together, our results show that in pretreatment experiments, DSF/Cu significantly inhibited the stemness of NCI-H1299 cells *in vitro*.

We also observed that DSF/Cu pretreatment of NCI-H1299 cells resulted in a dramatic decrease in tumor seeding relative to cells pretreated with DMSO (control), paclitaxel and DSF alone (Figure 4F and Table 1). The tumor incidence and latency period in mice receiving DSF/Cu-pretreated cells showed clear distinctions relative to mice receiving cells pretreated with DMSO, paclitaxel or DSF alone (Table 1). These findings confirmed that ALDH-positive CSCs within NSCLCs are resistant to paclitaxel and DSF alone, but are sensitive to DSF/Cu. Treatment with the DSF/Cu complex may be a potential strategy to inhibit NSCLC recurrence driven by ALDH-positive CSCs.

**Table 1:** Tumor incidence and latency period in xenograft mice receiving cells pretreated with different drugs

Group	Cells injected	Tumor incidence	Latency period(days)
Control	5000	6/6 (100.0%)	35
	1000	6/6 (100.0%)	57
	100	3/6 (50.0%)	56
Paclitaxel	5000	6/6 (100.0%)	48
	1000	5/6 (83.3%)	52
	100	2/6 (33.3%)	72
DSF	5000	7/7 (100.0%)	33
	1000	6/6 (100.0%)	54
	100	6/6 (100.0%)	62
DSF/Cu	5000	1/6 (16.7%)	88
	1000	0/7 (0.0%)	-
	100	0/6 (0.0%)	-

### Effects of DSF/Cu treatment *in vivo* and tumor regrowth on secondary xenografts

**Figure 5:**
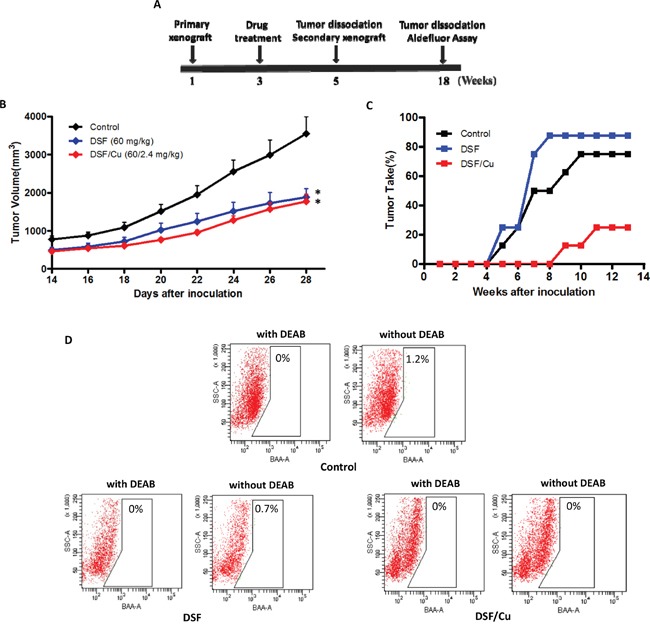
Effects of DSF and DSF/Cu treatment *in vivo* and tumor growth on secondary xenografts **A.** Schematic representation of the treatment scheme. **B.** Drug treatment reduces the size of primary xenograft tumors (*P < 0.05, 2-tailed *t* test). **C.** Percentage of tumor take and latency period in mice receiving secondary xenografts from each treatment group. **D.** Percentage of ALDH-positive cells in secondary xenograft tumors from each treatment group.

To determine whether DSF/Cu can target ALDH-positive cells and inhibit tumor growth and recurrence *in vivo*, we used a secondary xenograft model of NCI-H1299 cells in NOD/SCID mice (Figure 5A). Three groups of mice (NaCl solution control, DSF 60 mg/kg and DSF/Cu 60/2.4 mg/kg; eight mice in each group) were implanted with tumor cells. The animals were intravenously injected with 0.9% NaCl solution, DSF (DSF-loaded lipid emulsion) or DSF/Cu (Cu, intragastric administration) every two days. After 2 weeks of treatment, the volumes of all the primary xenograft tumors in the DSF/Cu and DSF treatment groups were significantly smaller than those in control animals (Figure 5B). However, the ability of residual cancer cells to initiate tumors upon reimplantation in secondary mice is a more definitive assay, so we examined the growth of secondary tumors in NOD/SCID mice inoculated with primary tumor cells obtained from the primary xenografts. The results showed that cancer cells from control and DSF-treated animals exhibited rapid tumor regrowth and a higher tumor take percentage. However, the cancer cells obtained from DSF/Cu-treated mice largely failed to produce any tumors in the recipient mice up to 8 weeks after implantation (Figure 5C and Table 2). By day 67, tumor cells derived from DSF/Cu-treated mice only caused two tumors out of eight inoculations, whereas the other tumor cells yielded tumors as early as days 42 to 54 (Table 2). At the end of the experimental period (13 weeks), tumors were isolated from the animals and the tumor cells were analyzed by Aldefluor assay. As shown in Figure 5D, DSF/Cu reduced the ALDH-positive population to 0%.

**Table 2:** Tumor incidence and latency period in mice receiving secondary xenografts

Group	Cells injected	Tumor incidence	Latency period(days)
Control	5000	6/8 (75.0%)	48
DSF/5000	5000	7/8 (87.5%)	42
DSF/Cu/5000	5000	2/8 (25.0%)	67

As shown in Figure 5C, the residual cancer cells from animals treated with DSF alone exhibited more rapid tumor regrowth and a higher tumor take percentage than the control group. A reasonable explanation for this is that DSF inhibited non-CSCs in the primary tumors, which may increase the proportion of CSCs in the residual cancer cells. After inoculating the same number of residual cancer cells into the secondary mice, it would therefore be expected that the DSF group would exhibit more rapid tumor regrowth and a higher tumor take percentage than the control group.

These results suggest that DSF/Cu is able to eliminate ALDH-positive NSCLC stem cells in primary xenografts, thereby abrogating the regrowth of tumors in secondary mice. Taken together with the *in vivo* Aldefluor assay results, these findings suggest that the DSF/Cu complex, rather than DSF alone, targets ALDH-positive NSCLC stem cells with high potency and inhibits tumor recurrence *in vivo*.

### ALDH1A1 plays a key role in the DSF/Cu-induced elimination of cancer stem cells

**Figure 6:**
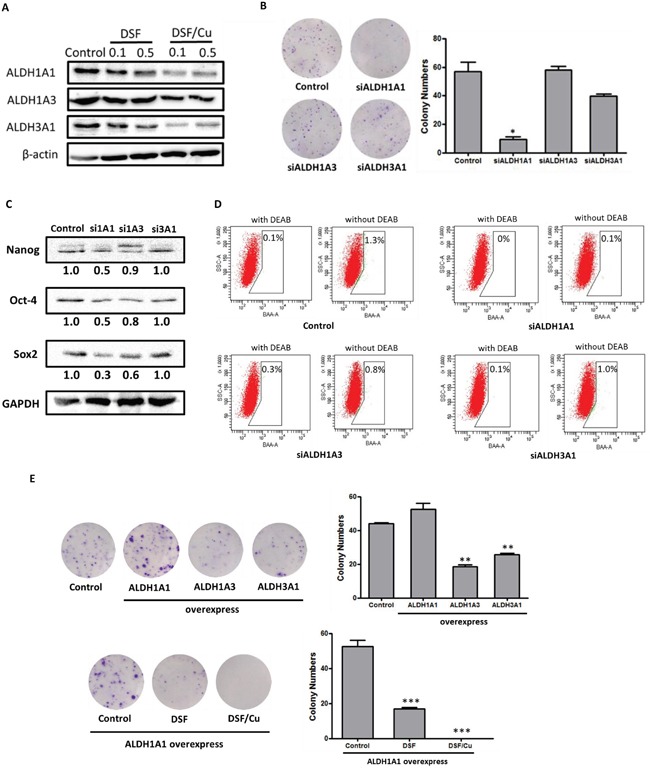
ALDH1A1 plays a key role in ALDH-positive NSCLC stem cells **A.** DSF (0.1 μM, and 0.5 μM) and DSF/Cu (0.1/1 μM, and 0.5/1 μM) inhibits the expression of the ALDH isozymes ALDH1A1, ALDH1A3 and ALDH3A1. **B.** The effects of siRNA knockdown of ALDH isozymes on colony-forming efficiency (*P < 0.05, one-way ANOVA). **C.** The levels of stem cell transcription factors in siRNA-treated cells. **D.** ALDH activity in siRNA-treated cells. **E.** Top row: the effects of overexpression of ALDH isozymes on the colony-forming efficiency of NCI-H1299 cells; bottom row: the inhibitory effects of DSF (0.5 μM) and DSF/Cu (0.5/1 μM) on ALDH1A1-overexpressing NCI-H1299 cells (**P < 0.01, ***P < 0.001, one-way ANOVA).

Recent evidence suggests that ALDH1 or ALDH3A1, which may be lung tumor stem cell markers or therapeutic targets, are highly expressed in some NSCLC cell lines as well as in patient lung cancer samples [21, 22]. However, the stem cell-related function and significance of the ALDHs have not yet been thoroughly investigated in NSCLCs. Therefore, we firstly examined the inhibitory effects of DSF, Cu and DSF/Cu treatment on the expression of three ALDH isozymes, ALDH1A1, ALDH1A3 and ALDH3A1. As shown in Figure 6A, DSF/Cu dramatically reduced the expression of all three ALDH isozymes in a concentration-dependent manner, and DSF/Cu was far more potent than DSF alone.

To explore the role of ALDH isozymes in ALDH-positive NSCLC stem cells and the mechanism underlying the activity of DSF/Cu, we used specific siRNAs to silence ALDH1A1, ALDH1A3 and ALDH3A1 in NCI-H1299 cells. We examined the effects of siRNA knockdown of ALDH isozymes on the capacity of the cells for self-renewal, the expression of stem cell transcription factors, and ALDH activity. Cells transfected with scramble siRNA were used as controls. The levels of ALDH isozymes in siRNA-treated cells were reduced by more than 70% compared with control cells (Supplemental Figure S8). We assessed the effects of knockdown of ALDH isozymes on colony-forming efficiency. As shown in Figure 6B, the colony numbers were significantly reduced by knockdown of ALDH1A1, but not of ALDH1A3 or ALDH3A1. This result suggests that ALDH1A1 predominantly contributed to the colony-forming capacity. Moreover, we assessed the expression of stem cell transcription factors in ALDH1A1, ALDH1A3 and ALDH3A1 knockdown cells. As shown in Figure 6C, the expression of Nanog, Oct-4 and Sox2 was more strongly reduced in ALDH1A1 knockdown cells than in ALDH1A3 or ALDH3A1 knockdown cells, suggesting that ALDH1A1 plays a key role in maintaining the stemness of NCI-H1299 cells. Furthermore, we examined the ALDH levels in siRNA-treated cells by Aldefluor assays, and found that ALDH1A1 and ALDH1A3 contribute to functional ALDH activity (Figure 6D). These results suggest that ALDH activity is associated with ALDH1A1 and ALDH1A3 expression, yet only ALDH1A1 plays a key role in maintaining ALDH-positive stem cells in NCI-H1299 cells.

To further evaluate whether ALDH1A1 is the target of the DSF/Cu complex, plasmids expressing human ALDH isozymes were transfected into NCI-H1299 cells. As shown in Figure 6E, ALDH1A1-overexpressing cells exhibited stronger colony-forming efficiency than normal NCI-H1299 cells. Interestingly, ALDH1A3- and ALDH3A1-overexpressing cells had lower colony-forming efficiency than control NCI-H1299 cells. Moreover, DSF and DSF/Cu both significantly inhibited the colony-forming capacity of ALDH1A1-overexpressing cells (Figure 6E). The inhibitory efficacy of DSF/Cu complex appeared to be stronger than that of DSF alone, but the difference was not statistically significant (Figure 6E).

Taken together, these results show that ALDH1A1, which plays a key role in maintaining ALDH-positive NSCLC stem cells, may be the target of the DSF/Cu complex.

## DISCUSSION

The anticancer efficacy of DSF, a clinically used anti-alcoholism drug, has been evaluated in various cancers [23–26]. DSF also enhances the cytotoxicity of several anticancer drugs as well as radiotherapy, suggesting that it is a potential chemotherapeutic agent [34]. An initial assessment of the effect of adding DSF to standard chemotherapy in lung cancer has recently been completed (NCT 00312819). The results showed that there was an increase in survival in the experimental group. Other Phase I/II clinical trials of DSF in melanoma and liver metastases (NCT00256230 and NCT00742911) are ongoing. However, the effect of DSF/Cu treatment in CSCs and its role as an irreversible inhibitor of ALDHs has not been thoroughly studied. Therefore, in the present study, both *in vitro* and *in vivo* systems were used to determine whether DSF acts against ALDH-positive NSCLC stem cells.

Previous reports have indicated that the anticancer activity of DSF is dependent on copper [26, 28–30]. Our data showed that CSCs within NSCLCs are resistant to paclitaxel and DSF alone but are sensitive to DSF/Cu (Figure 4–5). In other words, copper is critical for DSF-induced cytotoxicity in NSCLC stem cells. Mechanisms of copper-induced cytotoxicity are still poorly defined. It has been reported that bioavailable copper levels modulate tumor growth. Serum copper levels are elevated in cancer patients and copper-chelating drugs have antiangiogenic activity in animal models [35, 36]. Copper binding agents, which act as copper ionophores, lead to caspase inhibition and paraptotic cell death in human cancer cells [29]. DSF, as a bivalent metal ion chelator, forms a complex with copper and has enhanced cytotoxicity. In addition, DSF has low chemical stability in physiological environments [37]. The DSF/Cu complex may also improve the transport of DSF into cancer cells.

To explore whether the DSF/Cu complex may have an inhibitory effect on growth factor receptors mediating the expansion of CSCs, we tested the inhibitory effects of DSF and DSF/Cu treatment on the expression of EGFR, FGFR1 and IGF-1Rβ in both H1299 and A549 cells (Supplemental Figure S9). In the H1299 cell line, which contains ALDH-positive NSCLC stem cells, DSF (0.5 μM) and DSF/Cu (0.5/1 μM) greatly reduced the expression of EGFR, FGFR1 and IGF-1Rβ. The inhibitory effect of the DSF/Cu complex was stronger than that of DSF alone. However, in the A549 cell line, which does not contain ALDH-positive NSCLC stem cells, DSF/Cu decreased the protein levels of FGFR1 but not EGFR or IGF-1Rβ. These results suggest that by inhibiting ALDH, DSF and DSF/Cu may have an inhibitory effect on the expression of growth factor receptors that mediate the expansion of ALDH-positive CSCs.

In some cases, it has proved difficult to confirm markers that originally appeared to robustly distinguish tumorigenic from nontumorigenic cells [38]. Thus, it is necessary to identify markers that reproducibly distinguish tumorigenic and nontumorigenic cells, at least in specific subsets of experimental cell resources. Therefore, we used a serial implantation mouse model to investigate possible differences in the tumor formation potential of sorted ALDH-positive and ALDH-negative cells, which is the gold standard for determining CSCs (Figure 1). Our data support the hypothesis that ALDH may be a single CSC marker in some NSCLCs, regardless of the presence of other reported lung CSC markers, such as CD133, CD44 and so on. Nevertheless, the human ALDH superfamily comprises 19 isozymes and the different isozymes may possess multiple different catalytic and noncatalytic functions. Our data showed that only ALDH1A1 plays a key role in ALDH-positive NSCLC stem cells, suggesting that ALDH1A1 could be a potential target in clinical treatment (Figure 6).

Cancer stem cell theory partially explains tumor recurrence, drug resistance, and tumor metastasis. The emerging evidence linking stemness to poor prognosis and therapy failure suggests that therapeutic targeting of determinants of stemness might be an effective means to eradicate CSCs and prevent recurrence [39]. Thus, we used two NOD/SCID xenograft models to determine whether DSF/Cu can target ALDH-positive NSCLC stem cells and inhibit tumor recurrence *in vivo*, as assessed by tumor growth in recipient mice that were inoculated with tumor cells derived from DSF/Cu-treated cell lines or primary xenografts. The results showed that DSF/Cu complex treatment was able to target ALDH-positive NSCLC stem cells and inhibit tumor recurrence (Figures 4–5). We also observed that DSF/Cu pretreatment of H1299 cells resulted in a dramatic decrease in tumor-seeding ability relative to pretreatment with DMSO (control), paclitaxel and DSF alone (Figure 4F). These findings indicate that CSCs within NSCLC cell lines are resistant to paclitaxel and DSF alone but are sensitive to treatment with DSF/Cu.

In conclusion, we have shown that DSF/copper can inhibit ALDH-positive NSCLC stem cells *in vitro* and *in vivo*. These findings provide a strong rationale for clinical evaluation of the DSF/copper complex for lung cancer therapy.

## MATERIALS AND METHODS

### Cell lines and reagents

Human NSCLC cell lines (NCI-H460, NCI-H1299, NCI-H522 and A549) were obtained from the American Type Culture Collection. Authentication of these cell lines included morphology analysis, growth curve analysis and short tandem repeat analysis. Cells were routinely cultured in Roswell Park Memorial Institute 1640 medium supplemented with 10% fetal bovine serum (FBS) and maintained at 37°C in a humidified incubator with 5% CO2.

Disulfiram and Copper(II) D-gluconate were purchased from Sigma-Aldrich (St. Louis, MO, USA). The Disulfiram-loaded lipid emulsion was provided by Prof X Tang (Shenyang Pharmaceutical University, China). The drug loading content of the Disulfiram-loaded lipid emulsion was 3 mg/ml. The cumulative release of DSF from the lipid emulsion in 120 h was more than 60%. A pharmacokinetic study of DSF in rat plasma after intravenous administration of a dose of 36 mg/kg was carried out (t_1/2_ =0.1 h and t_1/2d_=0.3 h). The biological activity of the DSF-loaded lipid emulsion and conventional DSF has been compared previously [40].

The primary antibodies against Sox2, Oct-4 and Nanog were purchased from Cell Signaling Technology. Antibody to CD133/2 was purchased from Miltenyi Biotec. Antibodies to β-actin and GAPDH were obtained from Santa Cruz Biotechnology. Antibodies to ALDH1A1, ALDH1A3 and ALDH3A1 were obtained from Novus Biologicals. ALDH isozymes siRNA and Lipofectamine were obtained from Life Technologies. Human ALDH cDNA clone were obtained from Sino Biological Inc.

### Aldefluor assay and cell sorting

A cell population with a high ALDH enzyme activity was previously reported to be enriched in lung stem/progenitor cells [41]. Aldefluor assays were performed according to the manufacturer's guidelines (Stem Cell Technologies). Single cells obtained from cell cultures or xenograft tumors were incubated in Aldefluor assay buffer containing an ALDH substrate, bodipy-aminoacetaldehyde (1 μmol/l per 1,000,000 cells), for 30 to 60 min at 37°C. As a negative control, a fraction of the cells from each sample was incubated under identical conditions in the presence of the ALDH inhibitor diethylaminobenzaldehyde (DEAB). Flow cytometry was used to measure the ALDH-positive cell population. Desired cell populations were isolated using a FACSAriaIII flow cytometer (BD Biosciences).

### Colony forming assay

Cells were plated in 35-mm dishes at 300 cells/well and treated with different concentrations of drugs. Then the cells were incubated for an additional 7 to 12 days. Treatments were carried out in triplicate. The colonies obtained were fixed in formalin and stained with hematoxylin. The colonies were counted and compared with untreated cells.

### Tumorsphere formation assay

Single cells prepared from mechanical and enzymatic dissociation were seeded in 6-well ultra-low attachment plates (Corning, NY) at 3000 cells/well and cultured for about 2 weeks in serum-free DMEM/F-12 medium with B27 supplement (1×, Invitrogen), 20 ng/ml human recombinant bFGF (PeproTech), 20 ng/ml EGF (PeproTech), 10 ng/ml leukemia inhibitory factor (Chemicon) and 4 U/l insulin (Sigma).

### Western blotting analysis

Tumor tissue proteins were purified according to the reported method [42]. Equal amounts of total protein extracts from cultured cells or tissues were fractionated by 10–15% SDS-PAGE and electrically transferred onto polyvinylidene difluoride membranes (Bio-Rad, Richmond, CA). Mouse or rabbit primary antibodies and horseradish peroxidase (HRP)-conjugated appropriate secondary antibodies were used to detect the designated proteins. The bound secondary antibodies on the PVDF membrane were reacted with ECL detection reagents (Thermo Scientific) and exposed using an ImageQuant LAS 4000 mini system (GE Healthcare, Buckinghamshire, UK).

### Transwell invasion assay

Cellular potential for invasiveness was determined using Matrigel invasion chambers (BD Biosciences) according to the manufacturer's instruction. Briefly, cells were seeded into the upper chambers at 2×10^5^ per chamber in serum-free medium. The outer chambers were filled with the same medium but containing fetal bovine serum as the chemoattractant. Cells were incubated at 37°C for 24h, and the non-invading cells were removed by swabbing the top layer of the Matrigel. The cells were fixed and stained with Calcein-AM (Sigma-Aldrich). Chemotaxis was quantified with a high content drug screening system ImageXpressR Micro (Molecular Devices) by counting cells that had migrated to the lower side of the filter.

### Animal experiments

For *in vivo* identification of ALDH-positive NSCLC stem cells, ALDH-positive and ALDH-negative H1299 cells were sorted out by FACS and resuspended in 200μl 1:1 Matrigel:DMEM solution. Non-obese diabetic/severe combined immunodeficiency (NOD/SCID) mice were then inoculated with 5000 ALDH-positive cells in one flank and 5,000 ALDH-negative cells in the other flank. Tumor growth was allowed to proceed for 7 weeks and then the animals were humanely euthanized. Disassociated cells were obtained from the tumors and then were reanalyzed using the Aldefluor assay and sorted by FACS. Living cells from the dissociated tumors were sorted out by FACS for secondary xenografts. 500 or 5,000 ALDH-positive and ALDH-negative cells were inoculated in opposite sides of each NOD/SCID mouse. The growth of tumors was monitored and tumor volumes were measured every other day. Tumor volume was measured according to the equation: tumor volume = (π/6) × (L × W^2^), where L and W are the longer and shorter dimensions of the tumor. Mice were humanely euthanized when the larger one of the two tumors reached 800 to 1,000 mm^3^.

For *in vivo* drug treatment studies, ALDH-positive H1299 cells were sorted and resuspended in 200μl 1:1 Matrigel:DMEM solution, then 5,000 cells were inoculated into NOD/SCID mice. Two weeks after cell inoculation, animals were injected with DSF/Cu (30 mg/kg and 60 mg/kg) every other day for 5 weeks.

For drug pretreatment experiments, parental H1299 cells were treated with DMSO, paclitaxel (10 nM), DSF (0.5 μM) and DSF/Cu (0.5/1 μM) for 5 days and allowed to recover in the absence of drug for at least 5 days prior to injection *in vivo*. Cells from different groups were serially diluted (5×10^3^, 10^3^ and 10^2^) and then injected into NOD/SCID mice. Tumor incidence was monitored for 90 days after injection.

For *in vivo* drug treatment and secondary xenografts, 1×10^6^ H1299 cells were injected into NOD/SCID mice. Drug treatment (DSF 60 mg/kg, DSF/Cu 60/2.4 mg/kg) was initiated two weeks after injection. After two weeks of treatment, tumors were dissociated to a single-cell suspension for secondary xenografts. 5×10^3^ cells from each different group were inoculated into NOD/SCID mice. Tumor incidence was monitored for 90 days after injection.

Animal information: male, 18-22g, 4-5 week-old, NOD/SCID mice. This study was performed in strict accordance with the recommendations in the Guide for the Care and Use of Laboratory Animals of the National Institutes of Health. The protocol was approved by the Committee on the Ethics of Animal Experiments of Shenyang Pharmaceutical University.

### Dissociation of tumors

Mice were humanely euthanized and tumors were harvested. Tumor tissues were dissociated mechanically and enzymatically to obtain a single-cell suspension as previously described [3]. Briefly, tumors were minced by cutting with a scalpel and incubated in DMEM medium mixed with collagenase/hyaluronidase (Stem Cell Technologies) at 37°C for 6 hours. The tissues were further dissociated with 0.25% trypsin (Hyclone), dispase and DNase (Stem Cell Technologies), and then passed through a 40-μm nylon mesh to produce a single-cell suspension, which was used for Aldefluor assays and secondary xenografts.

### Immunohistochemistry

Tissues embedded in paraffin were cut into 4 μm-thick sections, deparaffinized, and treated with citrate buffer. The sections were then blocked with avidin/biotin for 20 min. The slides were incubated with primary antibody overnight at 4°C. Next, the slides were treated with secondary antibody with horseradish peroxidase goat anti-rabbit for 1–3 h and developed with 3, 3-diaminobenzidine (Sigma-Aldrich). Finally, the slides were counterstained with hematoxylin.

### RNA interference

RNA interference of ALDH subfamilies was done using short interfering RNA (siRNA; Invitrogen). A nonspecific scramble siRNA was used as control. For transfection, cells were seeded in 60 mm dishes and allowed to attach overnight. Cells were transfected with siRNA (20 nmol/l) using Lipofectamine (Invitrogen) according to the manufacturer's recommendations. The efficiency of siRNA was confirmed by western blot. 24 hours after transfection, the cells were collected and processed for analysis of colony forming assay, FACS and immunoblotting as described above.

### Overexpression of ALDH isozymes

Expression vectors were introduced into the NCI-H1299 cells by Sinofection (Sino Biological, Beijing, China). After 6 hours, the transfection mixture was removed and replaced by medium with serum. Colony forming assays were carried out after incubation for 72 hours. Cells were treated with DSF and DSF/Cu in the same manner as described above.

### Statistical analysis

Statistical software SPSS17.0 was used for all analyses. Data are presented as means ± standard error. Statistical differences were determined by two-tailed *t*-tests or by one-way analysis of variance for multiple comparisons. *P* values were derived using χ^2^ tests when the data didn't obey normal distribution. *P* values of <0.05 were considered statistically significant.

## SUPPLEMENTARY FIGURES AND TABLES



## References

[1] Reya T, Morrison SJ, Clarke MF, Weissman IL (2001). Stem cells, cancer, and cancer stem cells. Nature.

[2] Clarke MF, Dick JE, Dirks PB, Eaves CJ, Jamieson CH, Jones DL, Visvader J, Weissman IL, Wahl GM (2006). Cancer stem cells—perspectives on current status and future directions: AACR Workshop on cancer stem cells. Cancer Res.

[3] Al-Hajj M, Wicha MS, ito-Hernandez A, Morrison SJ, Clarke MF (2003). Prospective identification of tumorigenic breast cancer cells. Proc Natl Acad Sci U S A.

[4] Singh SK, Clarke ID, Terasaki M, Bonn VE, Hawkins C, Squire J, Dirks PB (2003). Identification of a cancer stem cell in human brain tumors. Cancer Res.

[5] Lee CJ, Dosch J, Simeone DM (2008). Pancreatic cancer stem cells. J Clin Oncol.

[6] Ricci-Vitiani L, Lombardi DG, Pilozzi E, Biffoni M, Todaro M, Peschle C, De Maria R (2007). Identification and expansion of human colon-cancer-initiating cells. Nature.

[7] Kelly K, Yin JJ (2008). Prostate cancer and metastasis initiating stem cells. Cell Res.

[8] Sullivan JP, Minna JD, Shay JW (2010). Evidence for self-renewing lung cancer stem cells and their implications in tumor initiation, progression, and targeted therapy. Cancer Metastasis Rev.

[9] Kim CF, Jackson EL, Woolfenden AE, Lawrence S, Babar I, Vogel S, Crowley D, Bronson RT, Jacks T (2005). Identification of bronchioalveolar stem cells in normal lung and lung cancer. Cell.

[10] Giangreco A, Groot KR, Janes SM (2007). Lung cancer and lung stem cells-strange bedfellows?. Am J Resp and Criti Care Med.

[11] Yoshida A, Hs u LC, Dave’ V (1992). Retinal oxidation activity and biological role of human cytosolic aldehyde dehydrogenase. Enzyme.

[12] Burger PE, Gupta R, Xiong X, Ontiveros CS, Salm SN, Moscatelli D, Wilson EL (2009). High aldehyde dehydrogenase activity: a novel functional marker of murine prostate stem/progenitor cells. Stem Cells.

[13] Ginestier C, Hur MH, Charafe-Jauffret E, Monville F, Dutcher J, Brown M, Jacquemier J, Viens P, Kleer CG, Liu S, Schott A, Hayes D, Birnbaum D, Wicha MS, Dontu G (2007). ALDH1 is a marker of normal and malignant human mammary stem cells and a predictor of poor clinical outcome. Cell stem cell.

[14] Huang EH, Hynes MJ, Zhang T, Ginestier C, Dontu G, Appelman H, Fields JZ, Wicha MS, Boman BM (2009). Aldehyde dehydrogenase 1 is a marker for normal and malignant human colonic stem cells (SC) and tracks SC overpopulation during colon tumorigenesis. Cancer Research.

[15] Lugli A, Iezzi G, Hostettler I, Muraro MG, Mele V, Tornillo L, Carafa V, Spagnoli G, Terracciano L, Zlobec I (2010). Prognostic impact of the expression of putative cancer stem cell markers CD133, CD166, CD44s, EpCAM, and ALDH1 in colorectal cancer. British journal of cancer.

[16] Carpentino JE, Hynes MJ, Appelman HD, Zheng T, Steindler DA, Scott EW, Huang EH (2009). Aldehyde dehydrogenase–expressing colon stem cells contribute to tumorigenesis in the transition from colitis to cancer. Cancer research.

[17] Prince ME, Sivanandan R, Kaczorowski A, Wolf GT, Kaplan MJ, Dalerba P, Weissman IL, Clarke MF, Ailles LE (2007). Identification of a subpopulation of cells with cancer stem cell properties in head and neck squamous cell carcinoma. Proc Natl Acad Sci U S A.

[18] Tanei T, Morimoto K, Shimazu K, Kim SJ, Tanji Y, Taguchi T, Tamaki Y, Noguchi S (2009). Association of breast cancer stem cells identified by aldehyde dehydrogenase 1 expression with resistance to sequential Paclitaxel and epirubicin-based chemotherapy for breast cancers. Clinical Cancer Research.

[19] Van den Hoogen C, Van der Horst G, Cheung H, Buijs JT, Lippitt JM, Guzmán-Ramírez N, Hamdy FC, Eaton CL, Thalmann GN, Cecchini MG, Pelger RC, van der Pluijm G (2010). High aldehyde dehydrogenase activity identifies tumor-initiating and metastasis-initiating cells in human prostate cancer. Cancer research.

[20] Visvader JE, Lindeman GJ (2008). Cancer stem cells in solid tumours: accumulating evidence and unresolved questions. Nature Reviews Cancer.

[21] Patel M, Lu L, Zander DS, Sreerama L, Coco D, Moreb JS (2008). ALDH1A1 and ALDH3A1 expression in lung cancers: correlation with histologic type and potential precursors. Lung Cancer.

[22] Jiang F, Qiu Q, Khanna A, Todd NW, Deepak J, Xing L, Wang H, Liu Z, Su Y, Stass SA, Katz RL (2009). Aldehyde dehydrogenase 1 is a tumor stem cell-associated marker in lung cancer. Mol Cancer Res.

[23] Lin J, Haffner MC, Zhang Y, Lee BH, Brennen WN, Britton J, Kachhap SK, Shim JS, Liu JO, Nelson WG, Yegnasubramanian S, Carducci MA (2011). Disulfiram is a DNA demethylating agent and inhibits prostate cancer cell growth. Prostate.

[24] Zhang H, Chen D, Ringler J, Chen W, Cui QC, Ethier SP, Dou QP, Wu G (2010). Disulfiram treatment facilitates phosphoinositide 3-kinase inhibition in human breast cancer cells *in vitro* and *in vivo*. Cancer Res.

[25] Morrison BW, Doudican NA, Patel KR, Orlow SJ (2010). Disulfiram induces copperdependent stimulation of reactive oxygen species and activation of the extrinsic apoptotic pathway in melanoma. Melanoma Res.

[26] Liu P, Brown S, Goktug T, Channathodiyil P, Kannappan V, Hugnot JP, Guichet PO, Bian X, Armesilla AL, Darling JL, Wang W (2012). Cytotoxic effect of disulfiram/copper on human glioblastoma cell lines and ALDH-positive cancer-stem-like cells. Br. J. Cancer.

[27] Ma I, Allan AL (2011). The role of human aldehyde dehydrogenase in normal and cancer stem cells. Stem Cell Reviews and Reports.

[28] Xu B, Shi P, Fombon IS, Zhang Y, Huang F, Wang W, Zhou S (2011). Disulfiram/copper complex activated JNK/c-jun pathway and sensitized cytotoxicity of doxorubicin in doxorubicin resistant leukemia HL60 cells. Blood Cells Mol Dis.

[29] Tardito S, Bassanetti I, Bignardi C, Elviri L, Tegoni M, Mucchino C, Bussolati O, Franchi-Gazzola R, Marchiò L (2011). Copper binding agents acting as copper ionophores lead to caspase inhibition and paraptotic cell death in human cancer cells. J. Am. Chem. Soc.

[30] Li Y, Fu SY, Wang LH, Wang FY, Wang NN, Cao Q, Wang YT, Yang JY, Wu CF (2015). Copper improves the anti-angiogenic activity of disulfiram through the EGFR/Src/VEGF pathway in gliomas. Cancer Letters.

[31] Yip NC, Fombon IS, Liu P, Brown S, Kannappan V, Armesilla AL, Xu B, Cassidy J, Darling JL, Wang W (2011). Disulfiram modulated ROS-MAPK and NF-κB pathways and targeted breast cancer cells with cancer stem cell-like properties. Br. J. Cancer.

[32] Duan L, Shen H, Zhao G, Yang R, Cai X, Zhang L, Jin C, Huang Y (2014). Inhibitory effect of Disulfiram/copper complex on non-small cell lung cancer cells. Biochem Biophys Res Commun.

[33] Monk M, Holding C (2001). Human embryonic genes re-expressed in cancer cells. Oncogene.

[34] Rae C, Tesson M, Babich JW, Boyd M, Sorensen A, Mairs RJ (2013). The role of copper in disulfiram-induced toxicity and radiosensitization of cancer cells. J. Nucl. Med.

[35] Donita Brady, Matthew Crowe, Michelle Turski, Hobbs G Aaron, Yao Xiaojie, Chaikuad Apirat (2014). Copper is required for oncogenic BRAF signalling and tumorigenesis. Nature.

[36] Garber K (2015). Cancer's copper connections. Science.

[37] Johansson B (1988). Stabilization and quantitative determination of disulfiram in human plasma samples. Clin Chim Acta.

[38] Jeffrey Magee, Elena Piskounova, Sean Morrison (2012). Cancer Stem Cells: Impact, Heterogeneity, and Uncertainty. Cancer Cell.

[39] Antonija Kreso, John Dick (2014). Evolution of the Cancer Stem Cell Model. Cell Stem Cell.

[40] Chen X, Zhang L, Hu X, Lin X, Zhang Y, Tang X (2015). Formulation and preparation of a stable intravenous disulfiram-loaded lipid emulsion. Eur. J. Lipid Sci. Technol.

[41] Jiang F, Qiu Q, Khanna A, Todd NW, Deepak J, Xing L, Wang H, Liu Z, Su Y, Stass SA, Katz RL (2009). Aldehyde dehydrogenase 1 is a tumor stem cell-associated marker in lung cancer. Mol Cancer Res.

[42] Li Y, Zhang T, Korkaya H, Liu S, Lee HF, Newman B, Yu Y, Clouthier SG, Schwartz SJ, Wicha MS, Sun D (2010). Sulforaphane, a Dietary Component of Broccoli/Broccoli Sprouts, Inhibits Breast Cancer Stem Cells. Clin Cancer Res.

