# Supplementary material for: Targeting ALDH1A1 by disulfiram/copper complex inhibits non-small cell lung cancer recurrence driven by ALDH-positive cancer stem cells
Source: Oncotarget. 2016 Aug 16;7(36):58516–30. doi: 10.18632/oncotarget.11305 (PMC5295448; doi:10.18632/oncotarget.11305)
Supplement: Supplementary file 1 [file oncotarget-07-58516-s001.pdf]

Targeting ALDH1A1 by disulfiram/copper complex inhibits non-small cell lung cancer recurrence driven by ALDH-positive cancer stem cells

SUPPLEMENTARY FIGURES

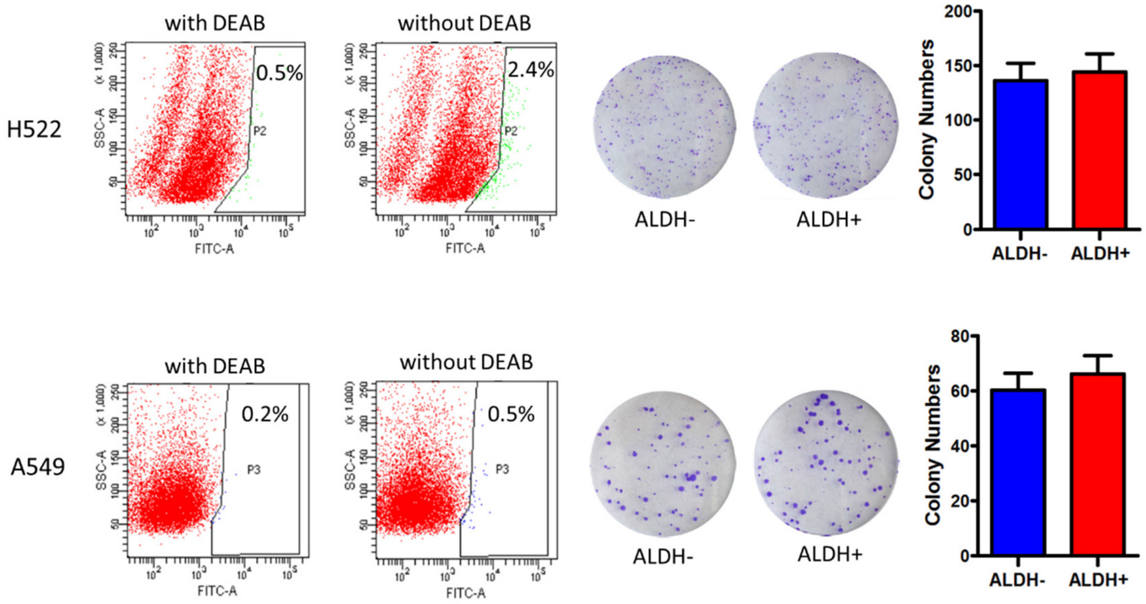

Supplementary Figure S1: Aldefluor assays and colony forming assays of ALDH-positive and ALDH-negative cells from the NCI-H522 and A549 cell lines.

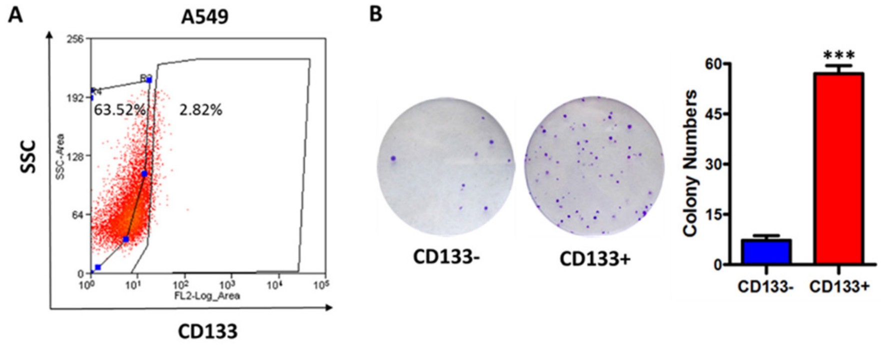

Supplementary Figure S2: CD133-positive cells represent cancer stem cells in A549 cells. A. CD133-positive and CD133-negative cells were isolated by FACS. **B.** Analysis of cell colony numbers in colony forming assays of CD133-positive and CD133-negative cells.

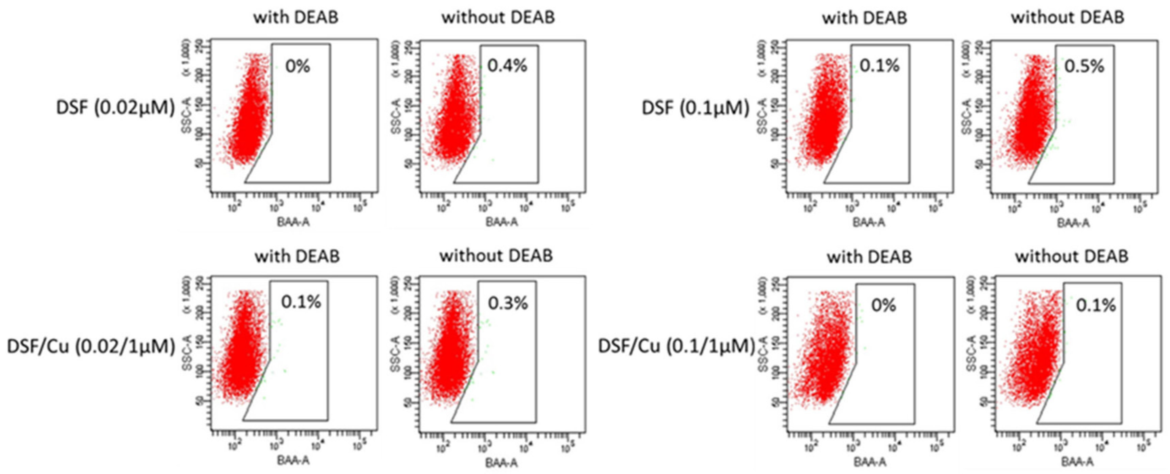

Supplementary Figure S3: DSF (0.02 μM, 0.1 μM) and DSF/Cu (0.02/1 μM, 0.1/1 μM) inhibit ALDH activity in NCI-H1299 cell lines.

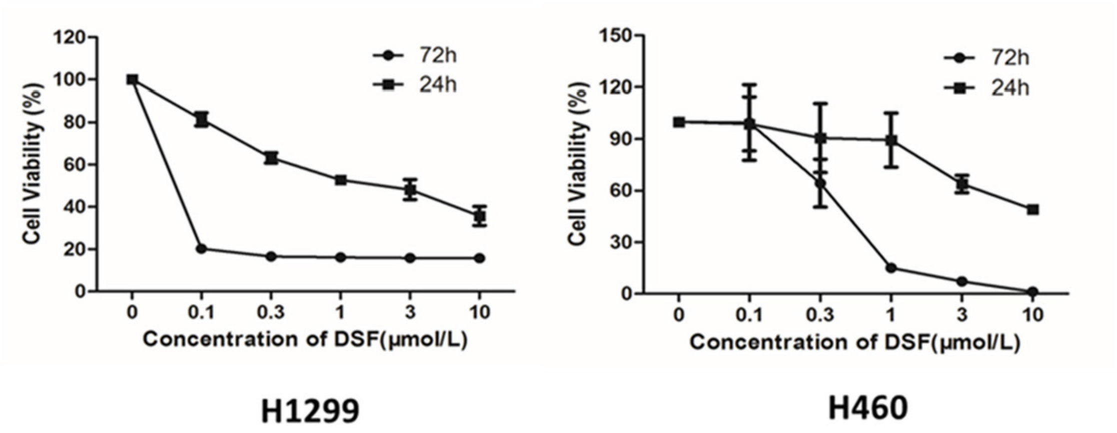

Supplementary Figure S4: DSF inhibits the proliferation of NCI-H1299 and NCI-H460 cells.

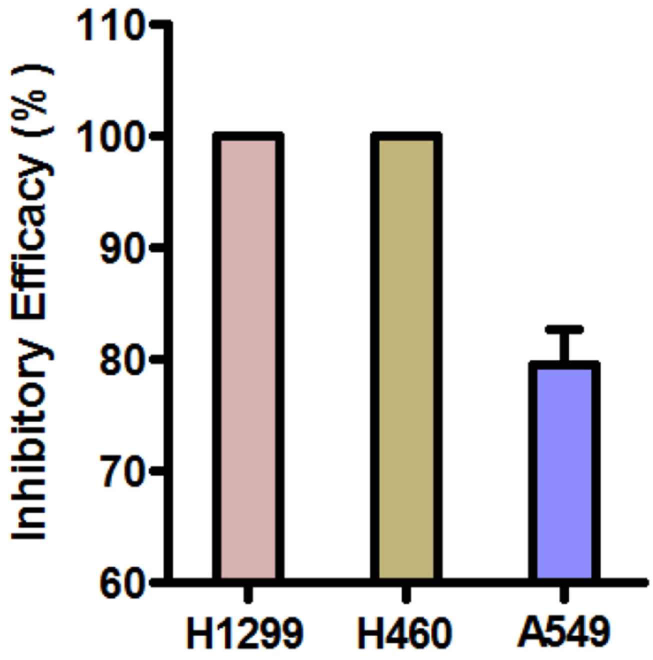

Supplementary Figure S5: Inhibitory efficacy of DSF/Cu in ALDH-positive NSCLC cells, as judged by colony forming assay.

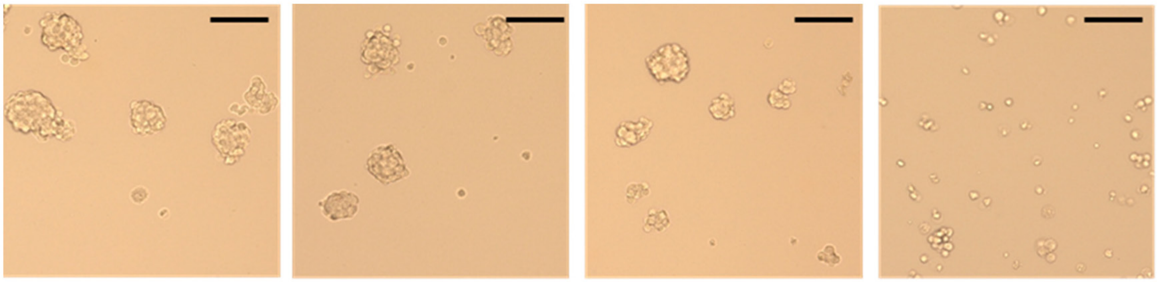

Supplementary Figure S6: Inhibitory effect of DSF and DSF/Cu on tumorsphere formation in NCI-H460 cells. (Scale bar, 100 µm)

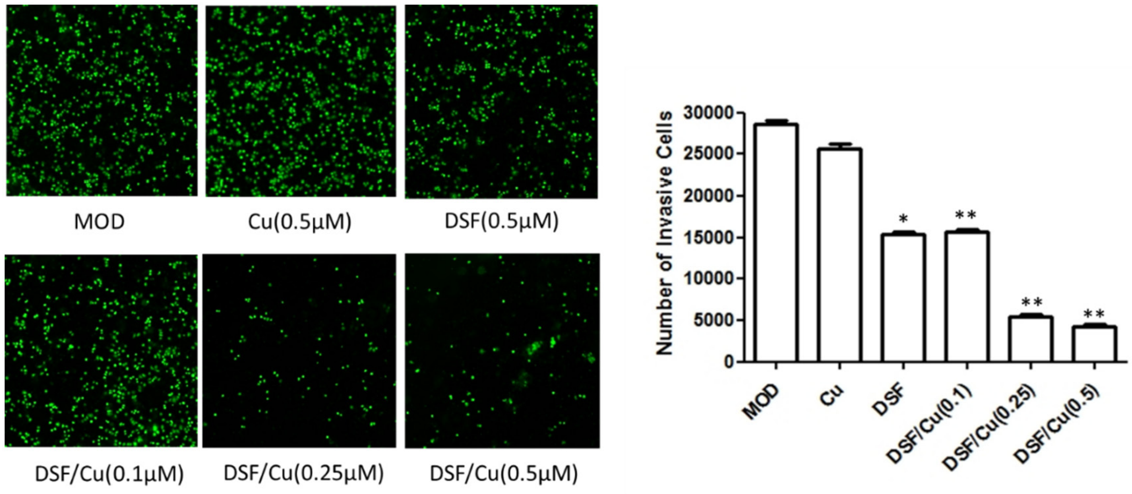

Supplementary Figure S7: DSF/Cu reduces the FBS-induced invasion of NCI-H460 cells.

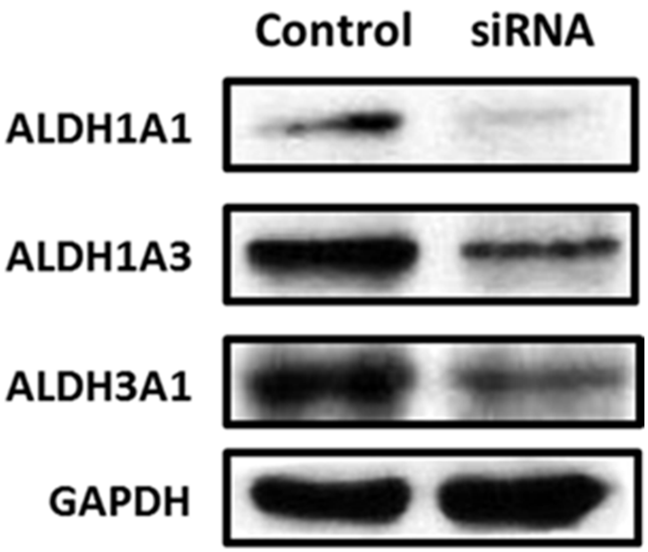

Supplementary Figure S8: The levels of ALDH isozymes in siRNA-treated NCI-H1299 cells.

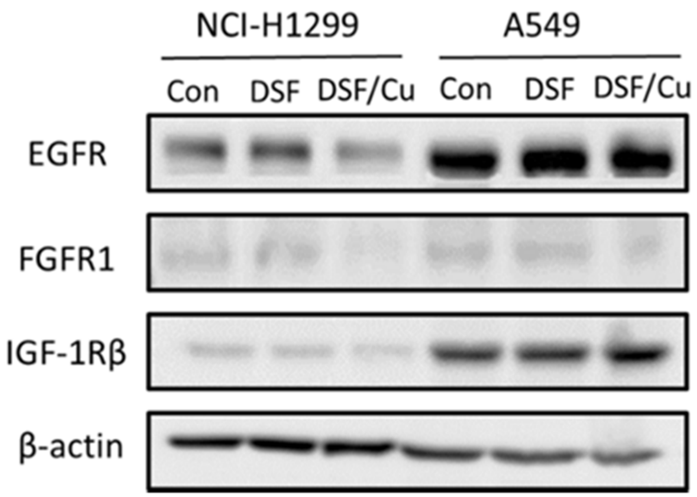

Supplementary Figure S9: Inhibitory effect of DSF (0.5 µM) and DSF/Cu (0.5/1 µM) treatment on the expression of EGFR, FGFR1 and IGF-1Rβ in H1299 and A549 cells.
